# Thermodynamic Stability Theories of Irreversible Processes and the Fourth Law of Thermodynamics

**DOI:** 10.3390/e26060442

**Published:** 2024-05-24

**Authors:** Vijay M. Tangde, Anil A. Bhalekar, Bjarne Andresen

**Affiliations:** 1 Department of Chemistry, Rashtrasant Tukadoji Maharaj Nagpur University, Nagpur 440 033, India; vijaytn6@gmail.com (V.M.T.); anabha@hotmail.com (A.A.B.); 2 Niels Bohr Institute, University of Copenhagen, Jagtvej 155 A, DK-2200 Copenhagen N, Denmark

**Keywords:** thermodynamic stability, nonequilibrium thermodynamics, Lyapunov stability theory, Gibbs–Duhem theories, the Fourth Law of thermodynamics, irreversible processes, rate of entropy production, multiple steady states

## Abstract

Three approaches for determining the thermodynamic stability of irreversible processes are described in generalized formulations. The simplest is the Gibbs–Duhem theory, specialized to irreversible trajectories, which uses the concept of virtual displacement in the reverse direction. Its only drawback is that even a trajectory leading to an explosion is identified as a thermodynamically stable motion. In the second approach, we use a thermodynamic Lyapunov function and its time rate from the Lyapunov thermodynamic stability theory (LTS, previously known as CTTSIP). In doing so, we demonstrate that the second differential of entropy, a frequently used Lyapunov function, is useful only for investigating the stability of equilibrium states. Nonequilibrium steady states do not qualify. Without using explicit perturbation coordinates, we further identify asymptotic thermodynamic stability and thermodynamic stability under constantly acting disturbances of unperturbed trajectories as well as of nonequilibrium steady states. The third approach is also based on the Lyapunov function from LTS, but here we additionally use the rates of perturbation coordinates, based on the Gibbs relations and without using their explicit expressions, to identify not only asymptotic thermodynamic stability but also thermodynamic stability under constantly acting disturbances. Only those trajectories leading to an infinite rate of entropy production (unstable states) are excluded from this conclusion. Finally, we use these findings to formulate the Fourth Law of thermodynamics based on the thermodynamic stability. It is a comprehensive statement covering all nonequilibrium trajectories, close to as well as far from equilibrium. Unlike previous suggested “fourth laws”, this one meets the same level of generality that is associated with the original zeroth to third laws. The above is illustrated using the Schlögl reaction with its multiple steady states in certain regions of operation.

## 1. Introduction

A sound thermodynamic theory of the stability of equilibrium states exists in the literature, based on Clausius inequalities and known as the Gibbs–Duhem thermodynamic theory of the stability of equilibrium states (see Glansdorff and Prigogine [1] and the recent analysis of the boundaries of thermodynamic stability [2,3,4]). Moreover, there is an equivalent De Donderian approach, also due to Prigogine (see Section XV of [5] or [6]) describing the stability of equilibrium states based on the direct use of the rate of entropy production as a positive sign-definite function that incorporates the effect of virtual displacement or perturbation in an unnatural direction.

This theory is indeed correct for equilibrium states but not when extrapolated to irreversible processes, e.g., steady states, as detailed by several examples further on. By contrast, a solid foundation for a general theory of stability is the famous Lyapunov’s second (direct) method of stability of motion [7,8,9,10,11,12,13,14,15,16,17] from 1892. This theory focuses on the trajectory (path) representing the ‘motion’ in question. Most frequently, it has been applied to physical trajectories, such as those from mechanics, hydrodynamics, aerodynamics, space science, etc., whose spatial coordinates are time dependent. For those, the environment as well as inaccurate internal controls of the motion of a ship, airplane, satellite, etc., may cause disturbances to the planned (unperturbed) trajectory. However, the theory is equally applicable to any other time-varying set of coordinates, e.g., thermodynamic coordinates, also without spatial dependence. In Lyapunov theory, one investigates the stability of not the individual transient nonequilibrium states making up the trajectory but the trajectory as a whole. Thus, in the domain of nonequilibrium thermodynamics, we need to identify trajectories in terms of time-dependent thermodynamic coordinates and then construct a sign-definite thermodynamic Lyapunov function. The only requirement for this function is that it vanishes only on the steady-state (i.e., unperturbed) trajectory.

Consequently, even though in nonequilibrium thermodynamics, one uses the term *local thermodynamic equilibrium* and correspondingly local equilibrium states, our focus cannot be on such individual states but on the trajectory composed of them. Importantly, the Lyapunov theory does not consist only of identifying a sign-definite function and evaluating the sign of its time variation. It investigates the stability of a given real trajectory, called an unperturbed trajectory. This stability analysis consists of the following four steps:

1. Generate perturbed trajectories by introducing sufficiently small perturbations (not necessarily along the original trajectory);
2. Define suitable thermodynamic perturbation coordinates, construct the corresponding thermodynamic perturbation space, and calculate the rate of change of the perturbation coordinates;
3. Identify a sign-definite thermodynamic Lyapunov function, dependent on the perturbation coordinates, that vanishes only on the unperturbed trajectory;
4. Determine the sign as well as the mathematical behavior of the variation with time of the chosen thermodynamic Lyapunov function.

Efforts by one of us (AAB) to follow all the steps of the Lyapunov theory resulted in the formulation of the Lyapunov thermodynamic stability (LTS) theory (previously named CTTSIP) [18,19,20]. Preliminary ideas were published recently [21]. There, we developed three generalized approaches. The first one is the Gibbs–Duhem-type thermodynamic stability theory for irreversible processes, which does not require the identification of a thermodynamic Lyapunov function but allows one to draw conclusions about the thermodynamic stability of irreversible trajectories. The other two generalized approaches are based on the LTS framework. In the first of them, no direct use of the equations of motion in terms of the perturbation coordinates is involved but still the most generalized deductions are arrived at. In the second approach, the rates of thermodynamic perturbation coordinates are defined via an appropriate Gibbs relation describing irreversible processes. Also, here, no detailed expression of the time variation of the thermodynamic perturbation coordinates is required. Hence, these three approaches to describing the thermodynamic stability of irreversible processes retain the generality element associated with thermodynamics and are complementary to one another.

Our thermodynamic Lyapunov function defined in Section 5, based on the rate of entropy production covering even far from equilibrium states, is a universal quantity that works very well in its generalized form as well as for individual cases. In essence, the LTS investigates to what extent a given irreversible trajectory is followed in spite of effected perturbations. This theory so far has been applied to several types of irreversible processes, including industrial (Haber–Bosch and contact processes), ecological, biological, and enzyme-catalyzed, and systems with multiple steady states, as well as some far from equilibrium (see e.g., [20,22,23,24,25,26,27,28] and other references on LTS cited therein). Our previous work did not attempt to develop a generalized description based on LTS. This aspect is of fundamental importance and will be worked out here. However, before we dig into the details of the LTS theory, we will set the scene by commenting on some previous attempts at achieving the desired generality but which fell short of full success.

Other papers have considered Lyapunov stability but only in approximate, specialized, or restricted situations, and never as a fully general formulation, e.g., the Lyapunov function of LTS resembles the one proposed earlier by Tarbell [29] for the study of the stability of nonequilibrium steady states of minimum entropy production. However, our Lyapunov function is capable of investigating all types of irreversible trajectories as well as time-invariant states. Our initial perturbation is a transient nonequilibrium state, which generates a perturbed trajectory whose fate with respect to the unperturbed trajectory is investigated. We also notice that since then, Hoang et al. [30,31], based on the LTS ideas but without referring to them, have suggested an alternate thermodynamic Lyapunov function, called a thermodynamic storage function, which is based on thermodynamic availability. They used Lyapunov’s second method to analyze the stability of continuously stirred tank reactors (CSTRs) [32]. Yet another proposal [33] used the perturbation coordinate itself as the Lyapunov function. Favache et al. [34] used Lyapunov’s first and second methods with the entropy, the entropy production, and the internal energy as possible Lyapunov functions on a CSTR system. As mentioned above, the rate of entropy production vanishes only in equilibrium states and thus is unsuitable for a general stability theory.

The central requirement of Lyapunov stability theory is that the chosen Lyapunov function must vanish *only* on the trajectory whose stability is under investigation (see Equation (13) of Section 4 and e.g., [15]). It seems that many authors on this subject so far have not taken this requirement to heart [1,6,33,34,35,36,37,38,39,40,41,42,43,44,45,46]. The result is that all previous attempts to define thermodynamic stability miss following all the steps of the Lyapunov second method, and many concentrate only on the stability of local *equilibrium* states (indeed, time-variant ones). That is not a correct approach. Admittedly, some of them do aim to investigate the stability of nonequilibrium steady states, but the chosen Lyapunov functions do not vanish at these nonequilibrium steady states.

For example, Glansdorff and Prigogine [1,35] proposed the second differential of the local equilibrium entropy $\delta^{2}s$ as a Lyapunov function, which indeed is sign-definite. Their condition of stability then is

(1) $$\begin{array}{c} \begin{array}{c} \delta^{2}s<0\;\mathrm{and}\;\frac{d}{dt}\left(\delta^{2}s\right)>0, \end{array} \end{array}$$

where close to equilibrium, $-\frac{1}{2}\delta^{2}s$ is a measure of the entropy production, a positive definite quantity (obviously for the local equilibrium state considered). It is also true that when a nonequilibrium state is very close to equilibrium, one can show the validity of the second inequality of Equation (1). However, that second differential vanishes only at an equilibrium state. Therefore, the conditions of Equation (1) determine the stability of the corresponding equilibrium state only. In view of this oversight, it is understandable that the conjecture of Landsberg [47] that Equation (1) is the statement of the Fourth Law of thermodynamics could not stand the test of time.

In the absence of realization of these basic facts, further works appeared in the literature. An example is the notable attempt by Seyfaie and Shaw [48], which extends the method to a trajectory generated by a sufficiently small perturbation of an equilibrium state and leading back to it. Such a trajectory passes through a succession of nonequilibrium states very close to equilibrium. Hence, it is clear that for such a trajectory, the inequalities of Equation (1) are satisfied. It seemingly constitutes a kind of stable motion. However, in the Lyapunov sense, it only establishes the asymptotic thermodynamic stability of the final *equilibrium* state because $\delta^{2}s$ vanishes only there (see the discussion in Section 4 and in particular Equation (13) for this implication).

Only a few years after the Glansdorff–Prigogine proposal was advanced, De Sabrino [43] analyzed to which degree it meets the requirements of Lyapunov’s direct method. This analysis pointed out its limitations, particularly for time-varying nonequilibrium states. In spite of that careful early analysis, an undeterred attempt to generalize the Glansdorff–Prigogine proposal appeared recently [44]. In the early years, Lavenda [49] developed a thermodynamic stability criterion based on system energy flows and applied it to linear and nonlinear phenomena, discussing its merits and limitations. Importantly, it also did not use Lyapunov’s second method in its entirety.

By contrast, recently, a novel and proper approach was suggested by Sieniutycz and Kuran [50], wherein they first proposed a sign-definite time rate of a Lyapunov function and then integrated it to arrive at the corresponding Lyapunov function (functional). Indeed, a Lyapunov function obtained this way vanishes only at the singular point (equilibrium or nonequilibrium) steady states, in conformity with the requirement of the second Lyapunov method of stability of motion. It involves the concept of the flux of perturbation coordinates in perturbation space and their driving forces defined via the gradients of corresponding Planck chemical potentials using Maxwell–Cattaneo-type constitutive equations. Indeed, this formulation is correct, but the form of the Lyapunov function obtained this way varies from system to system and with the approximations involved.

In the following, we first present a detailed development of our Lyapunov-type stability analysis of irreversible processes, specifically in terms of thermodynamic quantities. Then, in Section 10, we illustrate how LTS works in individual cases through a representative example of reactions in a continuously stirred tank reactor (CSTR), the Schlögl reaction. Finally in Section 11, we offer a new formulation of the Fourth Law of thermodynamics based on the present conclusions about the thermodynamic stability of irreversible trajectories. It encompasses the thermodynamic stability of *all* irreversible processes, including those far from equilibrium. All previous proposals for a fourth law of thermodynamics (see for example [1,51,52,53,54,55,56,57,58,59,60,61,62,63]) were centered around some specific physical aspect but lacked the generality associated with the previous established laws of thermodynamics (zeroth to third laws).

## 2. The Concept of Virtual Displacement

The concept of virtual displacement is used in the Gibbs–Duhem theory of the stability of equilibrium states [35,64,65,66]. Here, we extend it for determining the stability of irreversible processes. The thermodynamic description of a natural direction of irreversible evolution towards an equilibrium state of a system is described by the following inequalities [35,64,65,66]:

(2) $$\begin{array}{c} \begin{array}{c} {\left(\Delta S\right)}_{U,\;V}>0,\;{\left(\Delta A\right)}_{T,\;V}<0,\;{\left(\Delta G\right)}_{T,\;p}<0 \end{array} \end{array}$$

and

(3) $$\begin{array}{c} \begin{array}{c} {\left(dS\right)}_{U,\;V}>0,\;{\left(dA\right)}_{T,\;V}<0,\;{\left(dG\right)}_{T,\;p}<0, \end{array} \end{array}$$

where *S* is the entropy, *A* is the Helmholtz free energy, *G* is the Gibbs free energy, *U* is the internal energy, *V* is the volume, *T* is the temperature, and *p* is the pressure of the system.

Correspondingly, the unnatural direction of processes away from an equilibrium state is obviously described by the opposite inequalities, viz.,

(4) $$\begin{array}{c} \begin{array}{c} {\left(\Delta S\right)}_{U,\;V}<0,\;{\left(\Delta A\right)}_{T,\;V}>0,\;{\left(\Delta G\right)}_{T,\;p}>0 \end{array} \end{array}$$

and

(5) $$\begin{array}{c} \begin{array}{c} {\left(dS\right)}_{U,\;V}<0,\;{\left(dA\right)}_{T,\;V}>0,\;{\left(dG\right)}_{T,\;p}>0. \end{array} \end{array}$$

The unnatural direction described by Equations (4) and (5) may be translated into the impossibility of the stipulated changes described therein. Such stipulated changes are called *virtual displacements* in an unnatural direction. Hence, Equations (4) and (5) have been used as a formulation of the thermodynamic stability of equilibrium states. In other words, it means that *in the neighborhood of an equilibrium state, there exist equilibrium and nonequilibrium states that remain inaccessible under the respective conditions (4) and (5)*. Equivalently, these two sets of inequalities describe the impossibility of the envisaged virtual displacement.

## 3. Gibbs–Duhem Theory of Stability of Irreversible Processes

A part of this subject matter has been presented in the conference SIPS 2017 [21]. Recall that on an irreversible trajectory, the rate of entropy change is [1,5,21,67,68,69,70,71,72,73,74,75,76,77,78,79,80,81,82,83,84,85,86,87,88,89,90,91,92,93,94,95] (also refer to the review of various thermodynamic frameworks in [96]):

(6) $$\begin{array}{c} \begin{array}{c} \frac{dS}{dt}=\frac{d_{e}S}{dt}+\frac{d_{i}S}{dt}\;\mathrm{with}\;\frac{d_{i}S}{dt}\geq 0 \end{array} \end{array}$$

and at the local level

(7) $$\begin{array}{c} \begin{array}{c} \rho \frac{ds}{dt}+\nabla \cdot J_{s}=\sigma_{s}\geq 0, \end{array} \end{array}$$

where $d_{e}S≷0$ is the exchange-of-entropy differential, $d_{i}S>0$ is the entropy production differential, ρ is the mass density, *s* is the per unit mass entropy, $J_{s}$ is the entropy flux density, and $\sigma_{s}$ is the entropy source strength. A simpler version of Equation (7) is

(8) $$\begin{array}{c} \begin{array}{c} \frac{ds}{dt}=\frac{d_{e}s}{dt}+\frac{d_{i}s}{dt}\;\mathrm{with}\;\frac{d_{i}s}{dt}\geq 0. \end{array} \end{array}$$

Recall that Equations (6) and (8) are the descriptions of the forward motion on a given irreversible trajectory. That is, these equations can be taken as the prescription of the natural direction of an irreversible process.

The thermodynamic stability of an irreversible trajectory is determined by considering a *virtual displacement* in the reverse direction on the same trajectory. This we can ascertain by replacing *t* with −t. That is, it amounts to effecting the envisaged *virtual displacement* in the reverse direction. We see that by this time reversal, the time derivatives change as follows:

(9) $$\begin{array}{c} \begin{array}{c} \frac{dS}{dt}⟼\frac{dS}{d(-t)}=-\frac{dS}{dt},\;\frac{d_{e}S}{dt}⟼\frac{d_{e}S}{d(-t)}=-\frac{d_{e}S}{dt},\;\mathrm{but}\;\frac{d_{i}S}{dt}⟼\frac{d_{i}S}{d(-t)}\neq -\frac{d_{i}S}{dt}, \end{array} \end{array}$$

and at the local level, we have

(10) $$\begin{array}{c} \begin{array}{c} \frac{ds}{dt}⟼\frac{ds}{d(-t)}=-\frac{ds}{dt},\;\frac{d_{e}s}{dt}⟼\frac{d_{e}s}{d(-t)}=-\frac{d_{e}s}{dt},\;\mathrm{but}\;\frac{d_{i}s}{dt}⟼\frac{d_{i}s}{d(-t)}\neq -\frac{d_{i}s}{dt}. \end{array} \end{array}$$

Let us understand what Equations (9) and (10) convey:

1. The impossibility described by the envisaged third transformation in each one of them stems from the fact that the rate of entropy production cannot change its sign as per the second law of thermodynamics.
2. In view of the preceding fact, the first transformation in each one of them is also not possible, which demonstrates the nonconservative nature of the entropy function.
3. Thus, it is also demonstrated that the envisaged virtual displacement in the reverse direction on a given irreversible trajectory is impossible. This impossibility remains true from any position on the irreversible trajectory.

Therefore, in the Gibbs–Duhem sense, the trajectories described by Equations (6)–(8) are thermodynamically stable. This demonstration is on a generalized footing. The conclusion is valid for both types of segments of irreversible trajectories: (i) the one in which the rate of entropy production goes on decreasing, and (ii) the one in which the rate of entropy production goes on increasing.

As examples, we list below the cases of certain conditional evolutions. They are the ones whose final states are the corresponding equilibrium states. The following inequalities follow from Clausius’ inequality (refer to any standard textbook on physical chemistry or on thermodynamics):

(11) $$\begin{array}{c} \begin{array}{c} \begin{array}{c} {\left(\frac{dS}{dt}\right)}_{\mathrm{adiabatic}}={\left(\frac{d_{i}S}{dt}\right)}_{\mathrm{adiabatic}}\geq 0,\;{\left(\frac{d_{i}S}{dt}\right)}_{U,\;V}\geq 0,\\ {\left(\frac{d_{i}S}{dt}\right)}_{H,\;p}\geq 0,\;{\left(\frac{d_{i}S}{dt}\right)}_{T,\;V}\geq 0,\;{\left(\frac{d_{i}S}{dt}\right)}_{T,\;p}\geq 0. \end{array} \end{array} \end{array}$$

On effecting the *virtual displacement* in the reverse direction on the trajectories described by Equation (11), the inequalities should also become reversed, which is mathematically expressed as

(12) $$\begin{array}{c} \begin{array}{c} \begin{array}{c} {\left(\frac{dS}{dt}\right)}_{\mathrm{adiabatic}}={\left(\frac{d_{i}S}{dt}\right)}_{\mathrm{adiabatic}}\leq 0,\;{\left(\frac{d_{i}S}{dt}\right)}_{U,\;V}\leq 0,\\ {\left(\frac{d_{i}S}{dt}\right)}_{H,\;p}\leq 0,\;{\left(\frac{d_{i}S}{dt}\right)}_{T,\;V}\leq 0,\;{\left(\frac{d_{i}S}{dt}\right)}_{T,\;p}\leq 0. \end{array} \end{array} \end{array}$$

However, the second law of thermodynamics forbids this to happen. For chemically reacting spatially uniform systems, it can be shown that the inequalities of Equation (11), that is, under the condition of adiabaticity and constant U,V; H,p; T,V; and T,p, remain valid not only in the vicinity of equilibrium but also during the course of evolution from the initiation of reaction until the attainment of equilibrium. Therefore, the trajectories described by Equation (11) must be thermodynamically stable.

Now, consider the case of nonequilibrium steady states. The trajectories leading to a nonequilibrium steady state (for example, α and γ types in the Schlögl reaction, refer Figure 3 of Section 10) are obtained as stable ones because a virtual displacement from them is in the unnatural direction and hence impossible. Oppositely, the β type of steady states (of the Schlögl reaction, refer to Figure 3 in Section 10) are not attainable ones because the natural direction of an irreversible trajectory leads away from them. Therefore, in this case, the virtual displacement is directed towards the said nonequilibrium steady state, the unnatural direction; hence, they are thermodynamically unstable ones.

## 4. Stability of Motion in Terms of Lyapunov Functions

Herein, only the final results of the stability of motion in terms of the identified Lyapunov functions are described. For details, the reader may consult the original sources cited in this paper [7,8,9,10,11,12,14,15,16,17]. In Lyapunov theory, a trajectory and a steady state whose stability is being investigated are called an unperturbed trajectory and an unperturbed state, respectively.

Let L>0 be an identified Lyapunov function that depends on time and on the perturbation coordinates of motion $\left\{x_{i}\left(t\right)\right\}$. It vanishes only for $\left\{x_{i}\left(t\right)=0\right\}$:

(13) $$\begin{array}{c} \begin{array}{c} L\left(\left\{0\right\},t\right)=0\;\mathrm{and}\;L\left(\left\{x_{i}\left(t\right)\right\},t\right)>0,\;\forall \left\{x_{i}\left(t\right)\right\}\in D\setminus \left\{0\right\}, \end{array} \end{array}$$

where D stands for the domain of $\left\{x_{i}\left(t\right)\right\}$ including 0, the origin or unperturbed trajectory. That is, only on the unperturbed trajectory does *L* vanish. (The notations in the preceding equation are the standard ones used in set theory and will also be used in the following when required.) Then, the differential equations of motion are given by

(14) $$\begin{array}{c} \begin{array}{c} \begin{array}{cc} \frac{dx_{i}}{dt} & =F_{i}\left(x_{1}\left(t\right),\;x_{2}\left(t\right),\;x_{3}\left(t\right),\;\ldots ,x_{n}\left(t\right);\;t\right),\;\left(i=1,2,3,\ldots ,n\right)\\ \; & =F_{i}\left(\{x_{j}\left(t\right)\};t\right),\;\mathrm{with}\;\left\{x_{j}\left(t\right)\right\}\equiv \left(x_{1}\left(t\right),\;x_{2}\left(t\right),\;x_{3}\left(t\right),\;\ldots ,x_{n}\left(t\right)\right), \end{array} \end{array} \end{array}$$

where $F_{i}\left(\{x_{j}\left(t\right)\};t\right)$ may be linear or nonlinear differential equations of motion in terms of the variables $\left\{x_{j}\left(t\right)\right\}$.

The stability and instability of motion in terms of the Lyapunov function $L=L(\left\{x_{i}\left(t\right)\right\};t)>0$ are as follows:

1. *Stable motion* is described by
  (15) $$\begin{array}{c} \begin{array}{c} \begin{array}{cc} \; & L\left(\left\{x_{i}\left(t\right)\right\};t\right)\geq W\left(\left\{x_{i}\left(t\right)\right\}\right)\geq 0\\ & \mathrm{with}\;\frac{dL}{dt}=\frac{\partial L}{\partial t}+\sum_{i}\frac{\partial L}{\partial x_{i}}\frac{dx_{i}}{dt}\leq 0\;\;\forall \left\{x_{i}\left(t\right)\right\}\in D\setminus \left\{0\right\}, \end{array} \end{array} \end{array}$$
  where the continuous function *W* has strict minima at the coordinate origin, L({0};t)=W({0})=0. This implies that after a small lapse of time, beyond $t_{0}$, nonetheless, we have L≠0 but $\dot{L}=0$. That can happen in the vicinity of the origin because initially, *L* is assumed to be a decreasing function of time (cf. Equation (15)).
2. *Asymptotically stable motion* is described by
  (16) $$\begin{array}{c} \begin{array}{c} \begin{array}{cc} \; & L\left(\left\{x_{i}\left(t\right)\right\};t\right)\geq W\left(\left\{x_{i}\left(t\right)\right\}\right)\geq 0\\ & \mathrm{with}\;\frac{dL}{dt}=\frac{\partial L}{\partial t}+\sum_{i}\frac{\partial L}{\partial x_{i}}\frac{dx_{i}}{dt}<0\;\;\forall \left\{x_{i}\left(t\right)\right\}\in D\setminus \left\{0\right\}. \end{array} \end{array} \end{array}$$
  That is, asymptotic stability requires the vanishing of $\dot{L}$ only at the origin, whereas in the case of stability, it can vanish very close to the origin.
3. When a motion is asymptotically stable and if all $\partial L/\partial x_{i}$ are bounded in absolute value, then according to Malkin’s theorem [7,9], the motion is *stable under constantly operating small disturbances*. A physical example of motion under constantly acting disturbances is that of an air flight experiencing rough weather.
4. *Asymptotic exponential stability* is described by
  (17) $$\begin{array}{c} \begin{array}{c} L>0,\;\frac{dL}{dt}=-\kappa \times L\;⟹\;L\leq L\left(t_{0}\right)\times e^{-\kappa (t-t_{0})}, \end{array} \end{array}$$
  where $t_{0}$ is the time of the initial perturbation. Thus, we see that for positive decay constant κ,
  (18) $$\begin{array}{c} \begin{array}{c} L(t)⟶0\;\mathrm{as}\;t⟶\infty . \end{array} \end{array}$$
  Negative κ would result in divergence, i.e., instability of the trajectory. How rapidly the perturbed trajectory returns to the unperturbed one depends on the value of κ, which needs to be determined experimentally.
5. Chetayev’s theorem of *instability* of motion requires that (see, for example, page 39 of [12] and page 226 of [15])
  (19) $$\begin{array}{c} \begin{array}{c} \begin{array}{cc} \; & L\left(\left\{x_{i}\left(t\right)\right\};t\right)\geq W\left(\left\{x_{i}\left(t\right)\right\}\right)\geq 0\\ & \mathrm{with}\;\frac{dL}{dt}=\frac{\partial L}{\partial t}+\sum_{i}\frac{\partial L}{\partial x_{i}}\frac{dx_{i}}{dt}>0\;\;\forall \left\{x_{i}\left(t\right)\right\}\in D\setminus \left\{0\right\}. \end{array} \end{array} \end{array}$$

The above description of Lyapunov’s theory uses a positive definite Lyapunov function. However, generally, it only has to be a sign-definite function. Hence, one is free to identify an L<0 and, for the stability of motion, the condition is dL/dt≥0. That is, in the above description, we just need to replace < with > and ≤ with ≥ and vice versa.

## 5. Thermodynamic Stability Based on the Thermodynamic Lyapunov Function of LTS

In view of the requirements on a chosen Lyapunov function described in the preceding Section 4, we have identified in our “Lyapunov thermodynamic stability” (LTS) the *thermodynamic Lyapunov functions*, $L_{S}$ (at the global level) and $L_{s}$ (at the local level). The identification of our thermodynamic Lyapunov function for irreversible processes is a generalization of using the rate of entropy production as the thermodynamic Lyapunov function for equilibrium states. Hence, our identified Lyapunov function will reduce to the rate of entropy production for equilibrium states.

The following definitions result [18,19,20]:

(20) $$\begin{array}{c} \begin{array}{c} L_{S}\left(t\right)=\left(\Sigma_{S}\left(t\right)-\Sigma_{S}^{0}\left(t\right)\right)\geq 0\;\mathrm{for}\;t\geq t_{0}, \end{array} \end{array}$$

where we have from the second law of thermodynamics (cf. Equation (6)) the following inequalities:

(21) $$\begin{array}{c} \begin{array}{c} \Sigma_{S}=\frac{d_{i}S}{dt}\geq 0,\;\Sigma_{S}^{0}={\left(\frac{d_{i}S}{dt}\right)}^{0}\geq 0; \end{array} \end{array}$$

$t_{0}$ is the time of effecting the perturbation, and the superscript 0 denotes the quantity pertaining to the unperturbed trajectory whose thermodynamic stability is being investigated. Notice that the Lyapunov function $L_{S}$ of LTS Equation (20) may be termed an excess entropy production function in the perturbation space because it is a difference between two rates of entropy production and it depends only on the perturbation coordinates. The positive definite sign in Equation (20) stems from the fact that, for thermodynamic reasons, we are considering only perturbations whose effect increases the rate of entropy production. As stated at the end of the preceding Section 4, one could equally well define $L_{S}\leq 0$. As far as we are dealing with irreversible trajectories, that would also work very well (in this case $L_{S}⟶-\infty$ at instability). However, by specializing this expression for determining the stability of equilibrium states, the result would be $\Sigma_{S}\leq 0$, which is against the second law of thermodynamics. All perturbations of an equilibrium state must increase the rate of entropy production, hence our choice of direction of the inequality in Equation (20). Further, the definition of Equation (20) is not restricted to close to equilibrium states. It also covers far from equilibrium situations, and this Lyapunov function vanishes only on the unperturbed trajectory.

At the local level, we have

(22) $$\begin{array}{c} \begin{array}{c} \rho \left(t\right)L_{s}\left(t\right)=\left(\sigma_{s}\left(t\right)-\sigma_{s}^{0}\left(t\right)\right)\geq 0\;\mathrm{for}\;t\geq t_{0}, \end{array} \end{array}$$

where $\sigma_{s}^{0}\left(t\right)$ and $\sigma_{s}\left(t\right)$ are the entropy source strengths (cf. Equation (7)), respectively, on unperturbed and perturbed trajectories. Herein too, $L_{s}$ can be termed the excess entropy production defined in terms of its respective entropy source strength. This inequality in Equation (22) allows certain interior cells to have $L_{s}=0$ but $L_{s}≮0$ because, by assumption, we have $L_{S}\geq 0$ and the same property needs to be inherited by $L_{s}$. Another reason is that the perturbation coordinates are defined (cf. Equation (26)) at the local level for a spatially nonuniform system. A crucial property of $L_{S}$ and $L_{s}$ is that both vanish on the real trajectory. That is, $L_{S}^{0}=0$ and $L_{s}^{0}=0$ because $\Sigma_{S}$ becomes $\Sigma_{S}^{0}$ and $L_{s}$ becomes $L_{s}^{0}$ on the unperturbed trajectory. The definition of Equation (22) is also very general, applicable to far from equilibrium situations.

We have the following relation between $L_{S}$ and $L_{s}$:

(23) $$\begin{array}{c} \begin{array}{c} L_{S}\left(t\right)=\int_{V}\rho \left(t\right)L_{s}\left(t\right)\;dV\geq 0, \end{array} \end{array}$$

whereas the global rate of entropy production functions $\Sigma_{S}$ and $\Sigma_{S}^{0}$ is

(24) $$\begin{array}{c} \begin{array}{c} \Sigma_{S}\left(t\right)=\int_{V}\sigma_{s}\left(t\right)\;dV\geq 0,\;\Sigma_{S}^{0}\left(t\right)=\int_{V}\sigma_{s}^{0}\left(t\right)\;dV\geq 0. \end{array} \end{array}$$

In LTS, the thermodynamic coordinates of motion can be identified either in terms of thermodynamic fluxes appearing in the expression of entropy source strength, or the choice can be based on the suitable Gibbs relation, keeping in mind that irreversible thermodynamics constitutes an autonomous system of description, that is, all thermodynamic quantities are time dependent only through their thermodynamic variables. Let us represent these coordinates by $\left\{y_{i}\left(t\right)\right\}$ and correspondingly the thermodynamic differential equations of motion as

(25) $$\begin{array}{c} \begin{array}{c} \begin{array}{cc} \frac{dy_{i}}{dt} & =F_{i}\left(\{y_{i}\left(t\right)\}\right),\;\left(i=1,2,3,\ldots ,n\right)\\ \; & \;\mathrm{with}\;\left\{y_{i}\left(t\right)\right\}\equiv \left(y_{1}\left(t\right),\;y_{2}\left(t\right),\;y_{3}\left(t\right),\;\ldots ,y_{n}\left(t\right)\right). \end{array} \end{array} \end{array}$$

The sufficiently small perturbation coordinates $\left\{\alpha_{i}\left(t\right)\right\}$ are defined as

(26) $$\begin{array}{c} \begin{array}{c} \alpha_{i}\left(t\right)=∥y_{i}\left(t\right)-y_{i}^{0}\left(t\right)∥\leq \varepsilon >0,\;\left(i=1,2,3,\ldots ,n\right). \end{array} \end{array}$$

**Figure 1:**
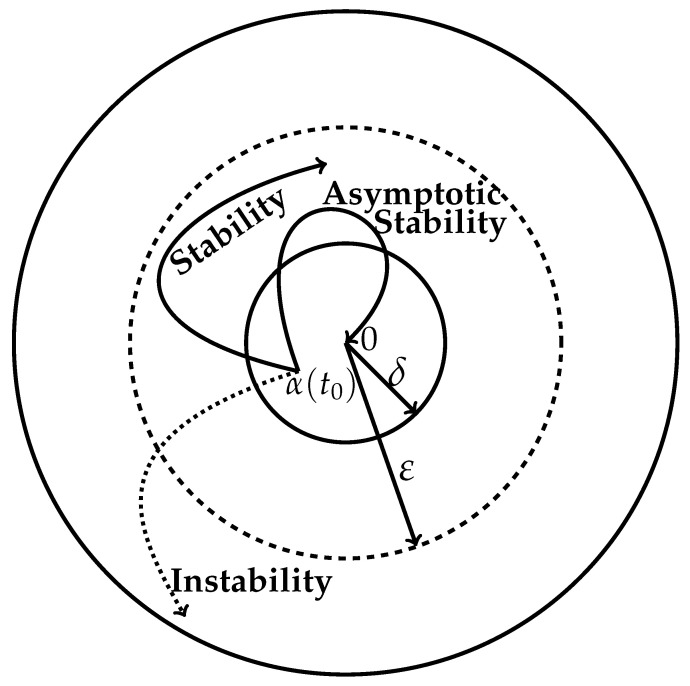
Schematic representation of an autonomous system of motion: stable, asymptotically stable, and unstable motions. A perturbation of magnitude $\alpha (t_{0})$ away from the unperturbed trajectory indicated as the origin 0 is effected at time $t=t_{0}$ within a sufficiently small region δ. If the perturbed trajectory remains within the region of magnitude ε, the real trajectory is said to be *stable*. If the perturbed motion within a short time tends back toward the unperturbed trajectory (the origin), the unperturbed motion is said to be *asymptotically stable*. And if the perturbed trajectory diverges from the region determined by ε, the motion is said to be *unstable*.

The superscript 0 refers to the unperturbed trajectory here and elsewhere. Obviously, the values of the initial perturbation coordinates are given by

(27) $$\begin{array}{c} \begin{array}{c} \alpha_{i}\left(t_{0}\right)=∥y_{i}\left(t_{0}\right)-y_{i}^{0}\left(t_{0}\right)∥\leq \delta \left(\varepsilon \right)>0,\;\left(i=1,2,3,\ldots ,n\right), \end{array} \end{array}$$

where δ and ε are the sufficiently small neighborhoods about the unperturbed trajectory as shown in Figure 1 such that δ<ε. The neighborhood δ about the unperturbed state 0 limits the magnitude of the initial sufficiently small perturbation. The neighborhood ε about the unperturbed state too is small but contains the δ region within it. Even if the perturbed trajectory does not enter into the δ region but does not cross out of the region ε, then the unperturbed motion still lies in close proximity to the unperturbed trajectory. Hence, the latter is a stable motion. In the case of asymptotic stability, the perturbed trajectory eventually tends back toward the unperturbed trajectory as depicted in Figure 1.

In this formulation, the $\alpha_{i}$ determine the perturbation space. Hence, the equations of unperturbed motion are

(28) $$\begin{array}{c} \begin{array}{c} \alpha_{i}^{0}\left(t\right)=0,\;\left(i=1,2,3,\ldots ,n\right),\;\mathrm{for}\;t\geq t_{0}. \end{array} \end{array}$$

On the Taylor expansion of Equation (25) in terms of the perturbation coordinates $\left\{\alpha_{i}\left(t\right)\right\}$, restricting the expressions to the linear terms only and using Equation (26), it is straightforward to obtain the following equations of motion in the perturbation space:

(29) $$\begin{array}{c} \begin{array}{c} \begin{array}{cc} \frac{d\alpha_{i}\left(t\right)}{dt} & =f_{i}\left(\{\alpha_{j}\left(t\right)\}\right),\;\left(i,\;j=1,2,3,\ldots ,n\right)\\ \; & \;\mathrm{with}\;\left\{\alpha_{j}\left(t\right)\right\}\equiv \left(\alpha_{1}\left(t\right),\;\alpha_{2}\left(t\right),\;\alpha_{3}\left(t\right),\ldots ,\alpha_{n}\left(t\right)\right). \end{array} \end{array} \end{array}$$

where the $f_{i}\left(\{\alpha_{j}\left(t\right)\};t\right)$ may be linear or nonlinear expressions in terms of the perturbation coordinates $\left\{\alpha_{j}\left(t\right)\right\}$.

For the sake of clarity, let us express the local level source strengths explicitly in terms of their thermodynamic coordinates,

(30) $$\begin{array}{c} \begin{array}{c} \sigma_{s}\left(t\right)=\sigma_{s}\left(\{y_{i}\left(t\right)\}\right),\;\sigma_{s}^{0}\left(t\right)=\sigma_{s}\left(\{y_{i}^{0}\left(t\right)\}\right). \end{array} \end{array}$$

Both are positive according to Equation (7). Then, the local thermodynamic Lyapunov function $L_{s}$ of Equation (22) is

(31) $$\begin{array}{c} \begin{array}{c} L_{s}\left(t\right)=L_{s}\left(\{\alpha_{i}\left(t\right)\}\right)>0\;\;\forall \left\{\alpha_{i}\left(t\right)\right\}\in D\setminus \left\{0\right\}, \end{array} \end{array}$$

where D stands for the domain of $\left\{\alpha_{i}\right\}$, that is, on the unperturbed trajectory

(32) $$\begin{array}{c} \begin{array}{c} L_{s}^{0}=L_{s}\left(0,\;0,\;0,\ldots ,0\right)=0\;\mathrm{for}\;t\geq t_{0}. \end{array} \end{array}$$

Therefore, the total time derivative of $L_{s}$ using Equations (29) and (31) is

(33) $$\begin{array}{c} \begin{array}{c} \frac{dL_{s}}{dt}=\sum_{i}\frac{\partial L_{s}}{\partial \alpha_{i}}\frac{d\alpha_{i}}{dt}=\sum_{i}\frac{\partial L_{s}}{\partial \alpha_{i}}f_{i}\left(\left\{\alpha_{i}\right\}\left(t\right)\right) \end{array} \end{array}$$

and that of $L_{S}$ is

(34) $$\begin{array}{c} \begin{array}{c} \frac{dL_{S}}{dt}=\int_{V}\rho \frac{dL_{s}}{dt}dV=\int_{V}\sum_{i}\rho \frac{\partial L_{s}}{\partial \alpha_{i}}f_{i}\left(\left\{\alpha_{i}\right\}\left(t\right)\right)\;dV. \end{array} \end{array}$$

Similarly, the perturbations leading to $L_{S}\leq 0$ ($L_{s}\leq 0$) can also be handled along the same lines. The details of the LTS tools have been described recently in [20].

The next step is to ascertain the signs (positive or negative) of Equations (33) and (34) using the signs of the involved partial derivatives and the time rates of perturbation coordinates $\left\{\alpha_{i}\left(t\right)\right\}$ defined using thermodynamic variables.

Notice a basic difference between the method of *virtual displacement in the reverse direction* and the one based on Lyapunov’s second method adopted in LTS. In the former, the impossibility of changing the positive sign of entropy production on the unperturbed trajectory is the central theme, while in LTS we analyze how the rate of entropy production on the perturbed trajectory varies with respect to that on the unperturbed trajectory.

In the following sections, we will discuss the possible consequences derived from the LTS approach.

## 6. Theorems of Thermodynamic Stability Using the Thermodynamic Lyapunov Function

Let us begin with the global-level thermodynamic Lyapunov function $L_{S}$ with the conditions and definition given in Equations (20) and (21). Now, as per the three perturbation curves of Figure 1, the time rate of $L_{S}$ has the following properties:

1. The *thermodynamic stability* of an unperturbed trajectory is guaranteed when we have
  (35) $$\begin{array}{c} \begin{array}{c} {\dot{L}}_{S}=\frac{dL_{S}}{dt}=\left(\frac{d\Sigma_{S}}{dt}-\frac{d\Sigma_{S}^{0}}{dt}\right)\leq 0. \end{array} \end{array}$$
  That is, the unperturbed motion described by Equation (28), i.e., $\alpha_{i}=0$, is stable. However, we see that in this way, the thermodynamic trajectories are obtained as merely stable. This is the case because mere stability means the perturbed trajectory finally settles in the close vicinity of the unperturbed one. This condition implies that
  (36) $$\begin{array}{c} \begin{array}{c} L_{S}=\Sigma_{S}-\Sigma_{S}^{0}=\mathrm{const}.\;\mathrm{with}\;\alpha_{i}\neq 0\;\;\mathrm{and}\;\;\alpha_{i}=\mathrm{const}.\;\;\left(i=1,2,3,\cdots ,n\right) \end{array} \end{array}$$
  is a possibility.
2. The *asymptotic thermodynamic stability* of an unperturbed trajectory follows whenever
  (37) $$\begin{array}{c} \begin{array}{c} {\dot{L}}_{S}=\frac{dL_{S}}{dt}=\left(\frac{d\Sigma_{S}}{dt}-\frac{d\Sigma_{S}^{0}}{dt}\right)<0\;\;\mathrm{except}\;\mathrm{when}\;\Sigma_{S}\to \Sigma_{S}^{0}. \end{array} \end{array}$$
  That is, ${\dot{L}}_{S}$ vanishes only when the perturbed trajectory reaches the unperturbed trajectory, making $L_{S}=L_{S}^{0}=0$, which happens only when all perturbation coordinates vanish.
3. Trajectories that are *asymptotically stable* as described by Equation (37) are, in view of Malkin’s theorem [7,9], also *thermodynamically stable under constantly acting perturbations* if, in addition, the derivatives
  (38) $$\begin{array}{c} \begin{array}{c} \frac{\partial L_{s}}{\partial \alpha_{i}}\;\left(i=1,2,3,\cdots ,n\right) \end{array} \end{array}$$
  at the local level are bounded in absolute value. Such constantly acting perturbations are, as said in the name, constantly being applied as opposed to the perturbations, leading to stability or to asymptotic stability, which are of a brief duration and then disappear, leaving the system to recover. This aspect of stability analysis using LTS is of practical use. For example, an industrial process runs continuously, and there is a possibility of repeated disturbances originating, say, due to the malfunctioning of heat exchangers to a reasonably small extent.
4. When a trajectory is *asymptotically stable* in the Lyapunov sense and additionally the following condition is obeyed,
  (39) $$\begin{array}{c} \begin{array}{c} \frac{dL_{S}}{dt}\leq -\kappa L_{S}\;\mathrm{implying}\;L_{S}\left(t\right)\leq L_{S}\left(t_{0}\right)e^{-\kappa (t-t_{0})}, \end{array} \end{array}$$
  then the unperturbed trajectory is said to be *exponentially stable*.
  Of course, in this case too, the result is $L_{S}=L_{S}^{0}$, which happens only when all perturbation coordinates vanish. How fast the perturbed trajectory would approach the unperturbed one is determined by the magnitude of κ, the rate constant of the exponential convergence.
5. Oppositely, according to Chetayev’s theorem [12,15], when
  (40) $$\begin{array}{c} \begin{array}{c} {\dot{L}}_{S}=\frac{dL_{S}}{dt}=\left(\frac{d\Sigma_{S}}{dt}-\frac{d\Sigma_{S}^{0}}{dt}\right)\;>\;0, \end{array} \end{array}$$
  the unperturbed trajectory is *thermodynamically unstable*.

It is pertinent to note at this juncture of our discussion that there is a difference between the stability in the Gibbs–Duhem sense and the Lyapunov sense. Gibbs–Duhem stability considers motion on a particular trajectory, forward and backward. Lyapunov stability considers any change in the trajectory on perturbation. Thus, the latter is a completely unrestricted and therefore more realistic approach.

## 7. Thermodynamic Stability of Irreversible Processes Using the Thermodynamic Lyapunov Function: A Generalized Description

The description of the present section does not use the explicit rates of perturbation coordinates $\alpha_{i}$. Notice that there may be three types of segments for irreversible trajectories. In one case, the rate of entropy production continuously increases with time but with decreasing steepness, thus approaching a new finite value. In the second type, the rate of entropy production continuously decreases with time and with decreasing steepness, again approaching a new finite value. A third type is where the rate of entropy production continuously increases, and the final result is a divergence. This latter case corresponds to a *thermodynamic instability* described in point 5 of the preceding Section 6 by Equation (40).

There are also cases where the rate of entropy production varies along a trajectory, for example, chemical oscillations and repetitive pattern formation (like Rayleigh–Bénard patterns). Those may be formed only when the transfer processes are nonlinear, e.g., due to nonlinear heat transfer, inertial effects, or chemical reactions involving several atoms of a particular kind. For asymptotically stable oscillating reactions, each segment of such reactions will be asymptotically stable, each one in its own direction, and the amplitude of the oscillations will decay as the original unperturbed trajectory is approached. The full mathematical formulation of such nonmonotonic trajectories turns out to be rather involved and will be presented separately in order not to dominate the presentation of the general Lyapunov thermodynamic stability theory (LTS) in the present paper.

An example of the chemical reaction type, the Schlögl reaction, is considered in Section 10. It offers two stable and one unstable steady-state branch (see Figure 3) as per Lyapunov’s first method. The second method of Lyapunov, i.e., LTS analysis of this system, reconfirms the above stability conclusions in a generalized way (see discussion of Section 10).

The first two types of stability/instability mentioned above fall in the category of *thermodynamic stability* because the decreasing steepness implies $d\Sigma_{S}^{0}/dt\geq 0$ and $d^{2}\Sigma_{S}^{0}/dt^{2}\leq 0$ for the first case and similarly with the inequalities flipped for the second case. Thus, the perturbed trajectory will eventually continue in very close proximity to the unperturbed trajectory. This implication is stated by Equation (36) of point number 1 of the preceding Section 6. In the present context, this means that eventually $d\Sigma_{S}/dt=d\Sigma_{S}^{0}/dt$ whether the perturbation coordinates $\alpha_{i}$ all vanish or not.

Since the conclusion includes asymptotic thermodynamic stability, it will also hold true for *exponential thermodynamic stability*. The corresponding value of κ of Equation (39) needs to be determined experimentally in individual cases.

For ascertaining *the thermodynamic stability under constantly acting disturbances*, we need to work out the sign and magnitude of each gradient of $L_{s}$ appearing in Equation (38) case by case. No generalized corresponding statement can be advanced.

In the case of *instability in the Lyapunov sense*, according to the Chetayev theorem [12,15] in Equation (40) we have $d\Sigma_{S}/dt>d\Sigma_{S}^{0}/dt$. That is, the rate of entropy production increases forever.

## 8. Thermodynamic Lyapunov Function-Based Generalized Thermodynamic Stability of Equilibrium and Nonequilibrium Steady States

The description of thermodynamic stability of equilibrium (eq) and of physically attainable nonequilibrium steady states (NSS), including NSS far from equilibrium, is generated simply by substituting $\Sigma_{S}^{0}=\Sigma_{S}^{eq}=0$ and $\Sigma_{S}^{0}=\Sigma_{S}^{NSS}=constant$, respectively, and keeping in mind that $\Sigma_{S}^{eq}$ and $\Sigma_{S}^{NSS}$ are time-independent quantities. Hence, in essence, we have *asymptotic thermodynamic stability* for equilibrium as well as physically attainable nonequilibrium steady states. Mathematically, it follows from Equation (37), which transforms to

(41) $$\begin{array}{c} \begin{array}{c} \frac{d\Sigma_{S}}{dt}<0. \end{array} \end{array}$$

This is accompanied by $\Sigma_{S}⟶0$ in the case of the asymptotic thermodynamic stability of an equilibrium state and by $\Sigma_{S}⟶\Sigma_{S}^{NSS}=constant\neq 0$ in the case of the asymptotic thermodynamic stability of a nonequilibrium steady state. It is easy to demonstrate that equilibrium as well as nonequilibrium steady states are thermodynamically stable under constantly acting disturbances. These results are all as expected.

Recall that evolution to reach a given NSS requires specific and appropriate conditions to drive the system; hence, such an evolution occurs under given conditions. Likewise, evolution to a final equilibrium state occurs under appropriate conditions on the system. For example, we may rewrite the conditional evolutions described in Section 3 by Equation (11) by including the time derivative of the corresponding rates of entropy production,

(42) $$\begin{array}{c} \begin{array}{c} \begin{array}{c} {\left(\frac{d_{i}S}{dt}\right)}_{\mathrm{adiabatic}}\geq 0,\;\frac{d}{dt}{\left(\frac{d_{i}S}{dt}\right)}_{\mathrm{adiabatic}}\leq 0\\ {\left(\frac{d_{i}S}{dt}\right)}_{U,\;V}\geq 0,\;\frac{d}{dt}{\left(\frac{d_{i}S}{dt}\right)}_{U,\;V}\leq 0 \end{array} \end{array} \end{array}$$

and

(43) $$\begin{array}{c} \begin{array}{c} \begin{array}{c} {\left(\frac{d_{i}S}{dt}\right)}_{H,\;p}\geq 0,\;\frac{d}{dt}{\left(\frac{d_{i}S}{dt}\right)}_{H,\;p}\leq 0,\\ {\left(\frac{d_{i}S}{dt}\right)}_{T,\;V}\geq 0,\;\frac{d}{dt}{\left(\frac{d_{i}S}{dt}\right)}_{T,\;V}\leq 0,\\ {\left(\frac{d_{i}S}{dt}\right)}_{T,\;p}\geq 0,\;\frac{d}{dt}{\left(\frac{d_{i}S}{dt}\right)}_{T,\;p}\leq 0. \end{array} \end{array} \end{array}$$

The involved conditions are adiabatic: constant U,V; H,p; T,V; T,p; etc.

In the case of nonequilibrium steady states, the resulting perturbation may also be such that the Lyapunov function is described as

(44) $$\begin{array}{c} \begin{array}{c} L_{S}=\left(\Sigma_{S}-\Sigma_{S}^{NSS}\right)\leq 0\;\mathrm{for}\;t\geq t_{0}, \end{array} \end{array}$$

which, for the reasons described in the preceding Section 6, leads to *asymptotic thermodynamic stability*, or mathematically

(45) $$\begin{array}{c} \begin{array}{c} \frac{d\Sigma_{S}}{dt}>0. \end{array} \end{array}$$

As an explicit example of the application of LTS, we have selected the Schlögl reaction [97], which is described in Section 10. The Schlögl reaction was designed as a simple model for bistable processes (see for example Chapter 8 of [88]). It also demonstrates that the physically unattainable nonequilibrium steady states are thermodynamically unstable (β-type of Figure 3).

It is interesting to recall that Lyapunov’s first method determines the stability of motion by solving the differential equations of motion. This approach does not require a Lyapunov function. However, the results obtained add substantially to our understanding of the system. For example, mathematically speaking, stationary state solutions are of two types, the stable and the unstable ones. Stable stationary points are attractive or neutral, i.e., small disturbances away from the stationary point will stay in the vicinity of that point. Unstable stationary points are repulsive, i.e., any small disturbance will move the system further away from stationarity. It is a constitutive theory approach. By comparison, LTS is a thermodynamic tool that does not use the ‘attractive’ and ‘repulsive’ terminology. Instead, it classifies stable, asymptotically stable, and unstable trajectories and likewise for the time-invariant states.

## 9. Using Thermodynamic Variables Based on Gibbs Relations and the Thermodynamic Lyapunov Function of LTS. A Generalized Account

For the sake of demonstration, we used the Gibbs relation of phenomenological irreversible thermodynamics, PIT (previously named GPITT) (see, for example, the recent publication [68]). The local-level Gibbs relation of PIT for a spatially nonuniform system is

(46) $$\begin{array}{c} \begin{array}{c} T\frac{ds}{dt}=\frac{du}{dt}+p\frac{dv}{dt}-\sum_{k,\;j}{\tilde{\mu }}_{k,\;j}\frac{d{\tilde{x}}_{k,\;j}}{dt}. \end{array} \end{array}$$

In this equation, instead of the traditional composition variables $\left\{x_{k}\right\}$ (mass fractions), we use the microscopic composition variables $\left\{{\tilde{x}}_{k,\;j}\right\}$ (mass fractions) and the corresponding chemical potentials {${\tilde{\mu }}_{k,\;j}\}$, where *j* denotes the quantum state of a molecule. The $\;\tilde{\;}\;$ over a symbol indicates that the quantity corresponds to the nonequilibrium population of quantum states. The rest of the symbols have their respective traditional meanings.

The functional dependence of entropy *s* from Equation (46) is

(47) $$\begin{array}{c} \begin{array}{c} s\left(t\right)=s\left(u\left(t\right),\;v\left(t\right),\;\{{\tilde{x}}_{k,\;j}\left(t\right)\}\right). \end{array} \end{array}$$

However, for the present purpose, we will use its equivalent representation:

(48) $$\begin{array}{c} \begin{array}{c} s\left(t\right)=s\left(T\left(t\right),\;p\left(t\right),\;\{{\tilde{x}}_{k,\;j}\left(t\right)\}\right). \end{array} \end{array}$$

Herein, we use the functional dependence of $L_{s}$ shown in Equation (31). Next, based on the functional dependence of Equation (48), we define the perturbation coordinates at the local level as

(49) $$\begin{array}{c} \begin{array}{c} \begin{array}{c} \delta T\left(t\right)\equiv ∥T\left(t\right)-T^{0}\left(t\right)∥,\;\delta p\left(t\right)\equiv ∥p\left(t\right)-p^{0}\left(t\right)∥,\;\\ \delta \left\{{\tilde{x}}_{k,\;j}\left(t\right)\right\}\equiv \left\{∥{\tilde{x}}_{k,\;j}\left(t\right)-{\tilde{x}}_{k,\;j}^{\;0}\left(t\right)∥\right\}. \end{array} \end{array} \end{array}$$

Now, the expression of Equation (33) is extended by using the expressions of Equation (49) to become

(50) $$\begin{array}{c} \begin{array}{c} \frac{dL_{s}}{dt}=\frac{\partial L_{s}}{\partial \delta T}\frac{d\delta T}{dt}+\frac{\partial L_{s}}{\partial \delta p}\frac{d\delta p}{dt}+\sum_{k,\;j}\rho \frac{\partial L_{s}}{\partial (\delta {\tilde{x}}_{k,\;j})}\frac{d(\delta {\tilde{x}}_{k,\;j})}{dt}\leq 0\;\;\forall \left\{\alpha_{i}\left(t\right)\right\}\in D\setminus \left\{0\right\}. \end{array} \end{array}$$

The negative time rate of $L_{s}$ in Equation (50) stems from the fact that, for a given condition of evolution, there is only one value of entropy source strength at a given point on the unperturbed trajectory under consideration. Hence, when its value on the perturbed trajectory by definition is instantly made higher than that on the unperturbed trajectory by way of the perturbation, the time rate of the former has to be such that the following inequality is followed:

(51) $$\begin{array}{c} \begin{array}{c} \frac{d\sigma_{s}}{dt}\leq \frac{d\sigma_{s}^{0}}{dt} \end{array} \end{array}$$

irrespective of whether the entropy source strength on the unperturbed trajectory has been increasing or decreasing with time. Thus, the mathematical statement of Equation (50) asserts the negative sign of the time rate of the Lyapunov function.

Now, the time rate of $L_{s}$ cannot vanish before reaching the unperturbed trajectory, because only then will we have equality in Equation (51), that is, when $\sigma_{s}=\sigma_{s}^{0}$. This is a consequence of the second law of thermodynamics. Thus, we see that the real trajectory must be *asymptotically stable*. Although the perturbation coordinates δT, δp, and $\left\{\delta {\tilde{x}}_{k,\;j}\right\}$ are positive by definition (cf. Equation (49)), the sign and magnitude of the gradients of $L_{s}$ of Equation (50) need to be worked out for each specific problem at hand. Hence, no generalized deduction about the *thermodynamic stability under constantly acting perturbations* can be stated herein.

Since the position of perturbation has not been restricted to be close to an end state (e.g., equilibrium and NSS), the above deduction is true in general. Notice that these deductions are the same as those presented in Section 7, demonstrating the consistency of the present discussion.

For determining how fast the perturbation would die out, one needs to work out individual cases for *exponential thermodynamic stability*. This type of thermodynamic stability is also guaranteed in view of the above deductions.

Moreover, from the local Equation (50), the expression of the time rate of the global Lyapunov function $L_{S}$ is obtained by spatial integration (cf. Equation (34)), yielding

(52) $$\begin{array}{c} \begin{array}{c} \begin{array}{c} \frac{dL_{S}}{dt}=\int_{V}\rho \frac{\partial L_{s}}{\partial \delta T}\frac{d\delta T}{dt}dV+\int_{V}\rho \frac{\partial L_{s}}{\partial \delta p}\frac{d\delta p}{dt}dV\;\;\;\;\\ +\int_{V}\sum_{k,\;j}\rho \frac{\partial L_{s}}{\partial (\delta {\tilde{x}}_{k,\;j})}\frac{d(\delta {\tilde{x}}_{k,\;j})}{dt}\leq 0\;\;\forall \left\{\alpha_{i}\left(t\right)\right\}\in D\setminus \left\{0\right\}. \end{array} \end{array} \end{array}$$

Herein, the domain D stands for $\left(\delta T,\;\delta p,\;\{\delta {\tilde{x}}_{k,\;j}\}\right)$. Thus, at the global level too, the real trajectories are found to fall in the *stable* category.

The above analysis can be easily specialized to the thermodynamic stability of equilibrium states and of nonequilibrium steady states. In the former case, we have $\sigma_{s}^{0}=0$, and in the latter it is $\sigma_{s}^{0}=const.$ That is, the time rate of change of $L_{s}$ is determined by the time rate of change of $\sigma_{s}$ pertaining to the respective perturbed trajectory. Moreover, the perturbation coordinates are defined for an equilibrium state as

(53) $$\begin{array}{c} \begin{array}{c} \begin{array}{c} \delta T\left(t\right)\equiv ∥T\left(t\right)-T^{eq}∥,\;\delta p\left(t\right)\equiv ∥p\left(t\right)-p^{eq}∥,\;\\ \delta \left\{{\tilde{x}}_{k,\;j}\left(t\right)\right\}\equiv \left\{∥{\tilde{x}}_{k,\;j}\left(t\right)-{\tilde{x}}_{k,\;j}^{\;eq}\left(t\right)∥\right\}\;\; \end{array} \end{array} \end{array}$$

and for a nonequilibrium steady state as

(54) $$\begin{array}{c} \begin{array}{c} \begin{array}{c} \delta T\left(t\right)\equiv ∥T\left(t\right)-T^{NSS}∥,\;\delta p\left(t\right)\equiv ∥p\left(t\right)-p^{NSS}∥,\;\\ \delta \left\{{\tilde{x}}_{k,\;j}\left(t\right)\right\}\equiv \left\{∥{\tilde{x}}_{k,\;j}\left(t\right)-{\tilde{x}}_{k,\;j}^{NSS}∥\right\}. \end{array} \end{array} \end{array}$$

We need to keep in mind that the perturbed trajectory corresponding to a nonequilibrium steady state evolves under the same overall conditions as the unperturbed nonequilibrium steady state. By contrast, in the case of an equilibrium state, we have several options. For example, if the perturbation does not create a spatial nonuniformity, then the conditions can be constancy of T,p or T,V or H,p or U,V, etc. And when the perturbation does generate spatial nonuniformity, the condition may be adiabaticity, isolation, or anything else that may be imposed on a spatially nonuniform system. It can be easily verified that the results would be identically the same as described in the preceding paragraphs.

## 10. Thermodynamic Stability Analysis of the Schlögl Reaction

**Figure 2:**
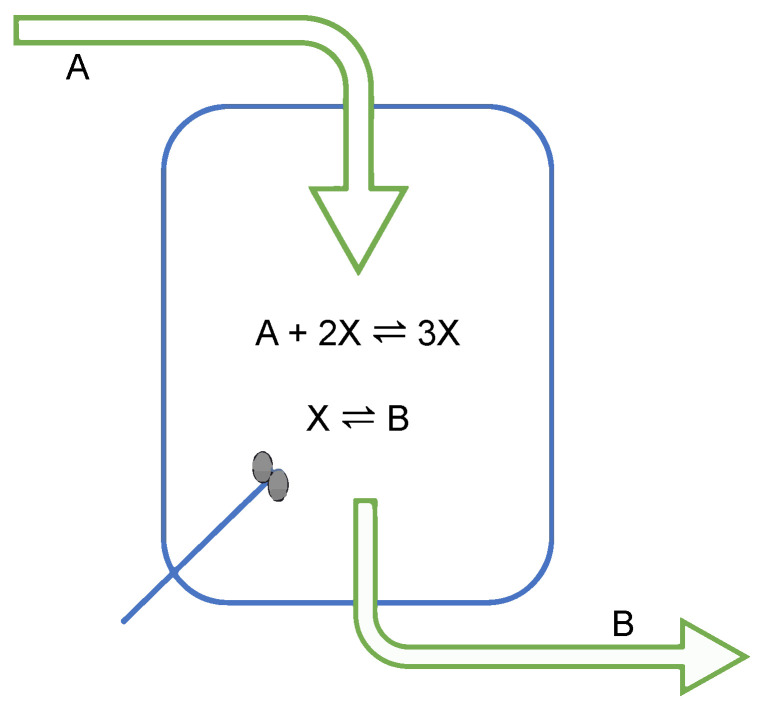
A schematic representation of the Schlögl reaction in a continuously stirred tank reactor (CSTR). The input of A and output of B are adjusted so that within the CSTR, they maintain constant concentrations $A^{0}$ and $B^{0}$, respectively. The intermediate component *X* is neither added nor withdrawn during the process. It adjusts itself as part of the reaction scheme.

In this section, we are applying LTS to a chemically reacting system in the steady state. As an illustrative example, we have chosen the standard Schlögl reaction in a continuously stirred tank reactor (CSTR) (Figure 2) [97,98]:

$$\begin{array}{c} \begin{array}{cc} \; & \mathrm{Reaction}I:\;A+2X\overset{k_{1}}{\underset{k_{2}}{⇌}}3X\\ \; & \mathrm{Reaction}\mathrm{II}:\;X\overset{k_{3}}{\underset{k_{4}}{⇌}}B \end{array} \end{array}$$

**Figure 3:**
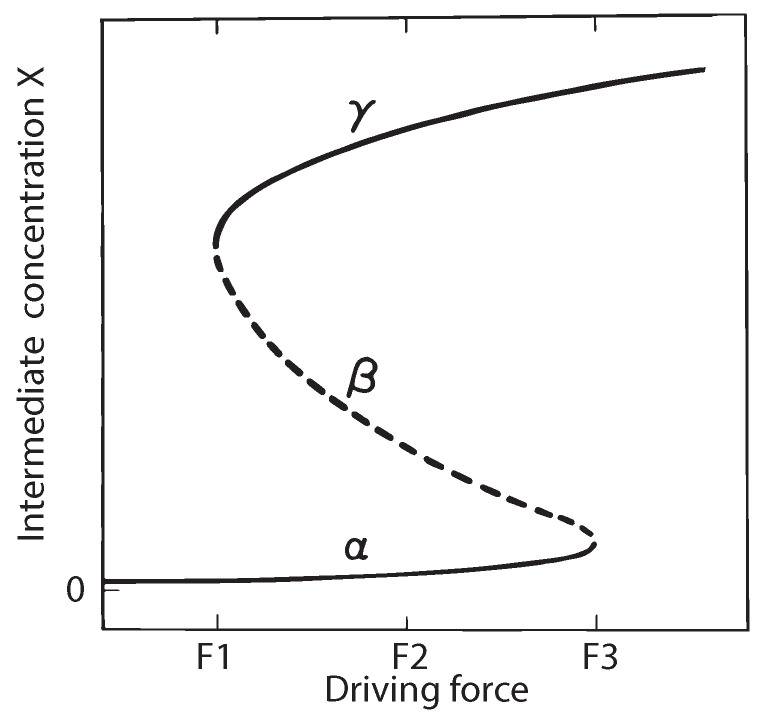
Steady states of the intermediate X of the Schlögl reaction are shown as the driving force (pump parameter) is increased. The solid parts of the curve are stable steady states, the dashed part is unstable.

The reason for choosing this reaction is that at low and high driving forces (primarily the concentration $A^{0}$), this pair of reactions has one stable steady state, while at intermediate driving force, it has three (two stable ones and one unstable); see Figure 3. The superscript 0 on concentrations in the following indicates the fixed value of that component in the CSTR. The inflow of A and the outflow of B are adjusted to maintain these values.

The rate of change of the concentration of X on an unperturbed trajectory denoted by the superscript 0 is

(55) $$\begin{array}{c} \begin{array}{c} \frac{dX^{0}}{dt}=k_{1}A^{0}{\left(X^{0}\right)}^{2}-k_{2}{\left(X^{0}\right)}^{3}-k_{3}X^{0}+k_{4}B^{0}. \end{array} \end{array}$$

Thus, at a steady state, we have for the concentration of X

(56) $$\begin{array}{c} \begin{array}{c} k_{1}A^{0}{\left(X^{0}\right)}^{2}-k_{2}{\left(X^{0}\right)}^{3}-k_{3}X^{0}+k_{4}B^{0}=0. \end{array} \end{array}$$

Solving this equation for $X^{0}$ yields either 3 real roots or 1 real root plus 2 complex conjugate complex roots. Taking the rate constants $k_{1}=k_{2}=k_{4}=1,\;k_{3}=4$, and $B^{0}=5$ in arbitrary units, we find that all concentrations $A^{0}$ result in only one steady state (a stable one), while $B^{0}=0.5$ shows three steady states in the region $3.9<A^{0}<8.3$ (two stable ones plus one unstable) as pictured in Figure 3 between the limiting points F1 and F3 (the particular concentrations $A^{0}$ driving the reaction between which the solution of Equation (56) is multivalued).

Notice that since we are considering nonequilibrium steady states (NSS) for the concentration of X, we have $X=X^{NSS}$. That is, the unperturbed trajectory is merely a time-independent point in the state space. On a perturbed trajectory, Equation (55) becomes

(57) $$\begin{array}{c} \begin{array}{c} \frac{dX}{dt}=k_{1}A^{0}X^{2}-k_{2}X^{3}-k_{3}X+k_{4}B^{0}, \end{array} \end{array}$$

where we have retained superscript 0 on A and B because the perturbation is effected only on X. In the present case, the expression of the rate of entropy production on a perturbed trajectory $\Sigma_{S}$ and at nonequilibrium steady state $\Sigma_{S}^{0}$ are

(58) $$\begin{array}{c} \begin{array}{c} \Sigma_{S}=\frac{A_{I}}{T}\frac{d\xi_{I}}{dt}+\frac{A_{\mathrm{II}}}{T}\frac{d\xi_{\mathrm{II}}}{dt}\geq 0,\;\Sigma_{S}^{0}=\frac{A_{I}^{0}}{T}\frac{d\xi_{I}^{0}}{dt}+\frac{A_{\mathrm{II}}^{0}}{T}\frac{d\xi_{\mathrm{II}}^{0}}{dt}\geq 0, \end{array} \end{array}$$

where the chemical affinities $A_{I}$, $A_{\mathrm{II}}$, $A_{I}^{0}$ and $A_{\mathrm{II}}^{0}$, in terms of chemical potentials are given by

(59) $$\begin{array}{c} \begin{array}{c} A_{I}=\mu_{A}^{0}-\mu_{X},\;A_{\mathrm{II}}=\mu_{X}-\mu_{B}^{0},\;A_{I}^{0}=\mu_{A}^{0}-\mu_{X}^{0},\;A_{\mathrm{II}}^{0}=\mu_{X}^{0}-\mu_{B}^{0} \end{array} \end{array}$$

and the $\xi_{i}$ and $\xi_{i}^{0}$ are the respective extents of advancement of each reaction.

Based on the general Equations (20), (21) and (35), which identify the excess rate of entropy productions as a Lyapunov function, the governing expressions of LTS for this set of reactions, in terms of a sufficiently small perturbation coordinate $\delta X=X-X^{0}$ are

(60) $$\frac{d(\delta X)}{dt}=\left(2k_{1}A^{0}X^{0}-3k_{2}{\left(X^{0}\right)}^{2}-k_{3}\right)\delta X$$

(61) $$L_{S}=\Sigma_{S}-\Sigma_{S}^{0}=\frac{A_{I}}{TX^{0}}\left[2\frac{d\xi_{I}^{0}}{dt}-k_{2}{\left(X^{0}\right)}^{3}\right]\delta X+\frac{A_{\mathrm{II}}}{T}k_{3}\delta X$$

(62) $$\begin{array}{cc} \; & \dot{L_{S}}=\frac{dL_{S}}{dt}=\frac{A_{I}}{TX^{0}}\left[2\frac{d\xi_{I}^{0}}{dt}-k_{2}{\left(X^{0}\right)}^{3}\right]\frac{d(\delta X)}{dt}\\ \; & \;\;\;+\frac{A_{\mathrm{II}}}{T}k_{3}\frac{d(\delta X)}{dt}-\frac{1}{T}\frac{\partial \mu_{X}}{\partial (\delta X)}{\left(\frac{d(\delta X)}{dt}\right)}^{2} \end{array}$$

and the gradient of $L_{S}$ in the perturbation space

(63) $$\begin{array}{c} \begin{array}{c} \frac{\partial L_{S}}{\partial (\delta X)}=\frac{A_{I}}{TX^{0}}\left[2\frac{d\xi_{I}^{0}}{dt}-k_{2}{\left(X^{0}\right)}^{3}\right]+\frac{A_{\mathrm{II}}}{T}k_{3}-\frac{1}{T}\left(\frac{\partial \mu_{X}}{\partial (\delta X)}\right)\frac{d(\delta X)}{dt}. \end{array} \end{array}$$

Here *R* is the universal gas constant and *T* is the temperature. The first term on the right-hand side of the second equality of Equations (61) and (62) as well as that of Equation (63) vanishes when the unperturbed state is a steady state for the concentration of X.

On using the values of rate constants described below Equation (56), the chemical affinities of reactions I and II become

(64) AI=AIO−RTlnXA0,XeqA0=k1k2=1∴AIO=0AII=AIIO−RTlnB0X,B0Xeq=k3k4=4∴AIIO=RTln4,

where AiO is the standard state affinity of the relevant reaction. In computing them, we have used that at equilibrium the chemical affinities $A_{I}=A_{\mathrm{II}}=0$ and used the second expressions of Equations (64) and (65) given by the laws of chemical kinetics. The above equations determine the net signs of $L_{S}$ and ${\dot{L}}_{S}$ and thus the stability of the steady-state solutions of (56) as derived by LTS.

**Figure 4:**
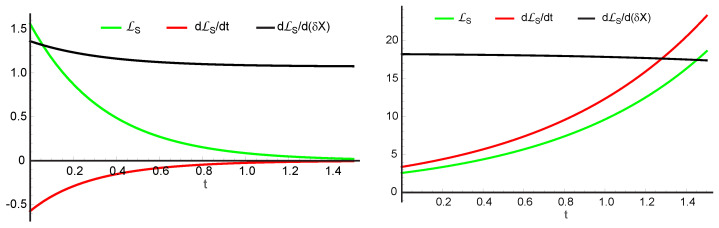
Evolution of the rate of excess entropy production $L_{S}$ (green), its time derivative $\dot{L_{S}}$ (red), and its gradient $\frac{\partial L_{S}}{\partial (\delta X)}$ (black) using rate constants $k_{1}=k_{2}=k_{4}=1,k_{3}=4$, and fixed concentrations $A^{0}=4$ and $B^{0}=0.5$ in the Schlögl reaction. The left graph is calculated around the first steady state point, a stable one. Here, the rate of excess entropy production $L_{S}$ is positive and its time rate is of the opposite sign throughout, both converging to zero. Thus, it is a case of *asymptotic thermodynamic stability*. Further, the gradient of $L_{S}$ remains finite throughout, making it also *thermodynamically stable under constantly acting disturbance*. The right graph is calculated around the second steady-state point, displaying divergent behavior and hence instability.

We illustrate this procedure by plotting the above equations with rate constants $k_{1}=k_{2}=k_{4}=1$, $k_{3}=4$, $A^{0}=4$, and $B^{0}=0.5$, i.e., for a case with three steady states. Solving Equation (56), we find that they are located at $X^{0}=0.14536$ (stable), $X^{0}=1.4030$ (unstable), and $X^{0}=2.4516$ (stable), corresponding to branches α, β, and γ of Figure 3, respectively. The left part of Figure 4 shows $L_{S},\dot{L_{S}}$, and $\frac{\partial L_{S}}{\partial (\delta X)}$ versus time resulting from the application of a 10% perturbation on *X* around the first steady state (α branch). We see that the excess entropy production $L_{S}$ and its rate of change $\dot{L_{S}}$ have opposite signs and both approach zero at long times. At the same time, the gradient of excess entropy production in perturbation space $\frac{\partial L_{S}}{\partial (\delta X)}$ remains finite. Thus, this is an *asymptotically stable* state as well as being *thermodynamically stable under constantly acting disturbances* according to Malkin’s theorem.

A similar set of curves results around the third steady state (γ branch) as well as for any set of parameters with only one steady state, e.g., the combination $k_{1}=k_{2}=k_{4}=1,k_{3}=4$, and $B^{0}=5$ mentioned earlier. By contrast, the results for the second steady state (β branch), also with a 10% perturbation on *X*, depicted in the right part of Figure 4 show the opposite behavior. There, the excess entropy production $L_{S}$ and its rate of change $\dot{L_{S}}$ have the same sign throughout and thus diverge away from the unperturbed state. In other words, they do not approach a steady state at long times. Also, the gradient of the excess entropy production in perturbation space, $\frac{\partial L_{S}}{\partial (\delta X)}$ eventually diverges. This is an unstable steady state and thus physically unattainable.

## 11. Formulating the “Fourth Law of Thermodynamics”

Historically, several candid proposals for the Fourth Law of thermodynamics have appeared. For example,

- The Onsager reciprocity relations (valid in the local thermodynamic equilibrium domain and resembling a constitutive theory) [51,52,53].
- Glansdorff–Prigogine’s proposal of Equation (1) (again, its domain of operation is close to equilibrium) [1,35].
- Maximum production of entropy [54,55,56,57,58] (note that Ross et al. [45] showed that this proposal does not stand the test of the second law of thermodynamics).
- Maximum rate of entropy production [59] or steepest entropy ascent [60] (however, it is not always true that all irreversible trajectories correspond to increasing entropy).
- An ecosystem selects the pathway that maximizes the free energy storage accompanying the organization of the state [61].
- A fourth law describing the nature of dark energy [62].
- The impossibility to design a Carnot engine or other physical heat engine whose source has a positive (absolute) temperature and its sink has a negative (absolute) temperature [63].

In view of the above claims, a question arises: What should be the precise object of the Fourth Law of thermodynamics? We believe that, considering the generality of the already established thermodynamic laws, the natural object of the missing Fourth Law must be the similar generality of the thermodynamic stability of irreversible processes, formulated such that it can even be applied to time-invariant states, viz., nonequilibrium steady states as well as equilibrium states.

Earlier, Landsberg rightly thought that the subject matter of the Fourth Law of thermodynamics should be thermodynamic stability, and then he conjectured the proposal of Glansdorff–Prigogine as its potential quantification [47]. But he erred by not realizing that taking the second differential of local entropy as a Lyapunov function amounts to describing the stability of only equilibrium states, as this second derivative vanishes only at equilibrium. That is why his suggestion of formulating the Fourth Law of thermodynamics in terms of the second differential of local equilibrium entropy as a universal thermodynamic Lyapunov function failed and could not stand the test of time.

In the analysis earlier in this paper, we concluded that as long as the rate of entropy production does not tend to infinity, neither on unperturbed nor on perturbed trajectories, the irreversible trajectory is thermodynamically stable in the Lyapunov sense. Therefore, we hereby propose the following formulation of the ‘Fourth Law of thermodynamics’.

$$\begin{array}{l} \mathrm{All}\;\mathrm{irreversible}\;\mathrm{processes}\;\mathrm{describable}\;\mathrm{globally}\;\mathrm{by}\\ \;\;\;\;\;\frac{dS}{dt}-\frac{d_{e}S}{dt}=\frac{d_{i}S}{dt}\geq 0\;(\mathrm{but}\;\mathrm{not}\;\mathrm{tending}\;\mathrm{to}\;\mathrm{infinity}\;\mathrm{in}\;\mathrm{Lyapunov}\;\mathrm{analysis})\\ \mathrm{and}\;\mathrm{at}\;\mathrm{the}\;\mathrm{local}\;\mathrm{level}\;\mathrm{by}\\ \;\;\;\rho \frac{ds}{dt}+\nabla \cdot J_{s}=\sigma_{s}\geq 0\;(\mathrm{but}\;\mathrm{not}\;\mathrm{tending}\;\mathrm{to}\;\mathrm{infinity}\;\mathrm{in}\;\mathrm{Lyapunov}\;\mathrm{analysis})\\ \mathrm{constitute}\;\mathrm{stable}\;\mathrm{thermodynamic}\;\mathrm{processes}\; \end{array}$$

In words, it can be stated as follows: *Stable and asymptotic thermodynamically stable processes cannot have unbounded values of their rates of entropy production even after perturbation in the Lyapunov sense*. Such processes, too, cannot be reversed.

Notice that LTS describes the thermodynamic stability of an entire trajectory right from the start, far from equilibrium, to the final state, whether that is an equilibrium state or not (cf. the discussion in Section 8). Hence, the uniqueness of LTS lies in its comprehensive approach to the task at hand, without the requirement that the system be close to equilibrium along the path. With the definition of $L_{S}$ in Equation (20), LTS tells us that $\Sigma_{S}^{0}=0$ when the unperturbed states are equilibrium states and $\Sigma_{S}^{0}$ is constant when the unperturbed states are nonequilibrium steady states. Nonequilibrium steady states far from equilibrium are also covered.

The zeroth law (reflexivity of equilibrium between systems), the first law (energy conservation), the second law (entropy of the universe increases), and the third law (entropy of a perfectly ordered structure at 0 K is 0) are all completely universal: they cover all situations, there are no exceptions, and there are no assumptions. The present version of the Fourth Law of thermodynamics as stated and explained above, matches the zeroth to third laws of thermodynamics in terms of its generality. Further, it complements their description of thermodynamics by providing a solid mathematical description of thermodynamic stability of irreversible trajectories.

## 12. Conclusions

In this paper, we have presented two existing theories of the thermodynamic stability of irreversible processes. The first, more traditional one, we have called a Gibbs–Duhem-type thermodynamic theory of the stability of irreversible processes. It is based on the concept of *virtual displacement in the reverse direction* on the real trajectory whose thermodynamic stability is under investigation. The characteristic of this theory is that the discussion remains on a generalized level. The only drawback of this theory is that it also classifies those trajectories leading to divergence (explosion) as thermodynamically stable.

This problem is solved by using instead the thermodynamic Lyapunov function of the theory of ‘Lyapunov thermodynamic stability’ (LTS). Our discussion here is on a more generalized level than what has been presented in our previous writings. The first approach presented above does not require the explicit use of perturbation coordinates. Still, we arrive at the *asymptotic thermodynamic stability of real trajectories describable by Equations (6) and (7). In this approach, we further demonstrate the* thermodynamic stability under constantly acting perturbations. It clearly shows that trajectories leading to an infinite value of rate of entropy production are correctly classified as *thermodynamically unstable*.

An illustrative demonstration of *thermodynamic stability under constantly acting perturbation* is one wherein we use the perturbation coordinates but not the explicit expressions of the differential equations of these perturbation coordinates. In this way, the approach still maintains its generalized characteristics. We have illustrated this approach using the Gibbs relation of ‘phenomenological irreversible thermodynamics’ (PIT) [68]. The Gibbs relation of other thermodynamic frameworks can also be used easily to reach the same conclusions. We emphasize that in the discussion of LTS, nowhere have we imposed a condition of close to or moderately away from equilibrium. Hence, the present investigation using LTS is valid even for far from equilibrium situations.

Finally, we have proposed a statement of the ‘Fourth Law of thermodynamics’ based on the thermodynamic stability analysis presented herein. It describes the thermodynamic stability of all irreversible trajectories, close to and far from equilibrium states, and in this sense it meets the criterion of generality associated with the existing laws of thermodynamics (the zeroth to third laws of thermodynamics). The same is not true for previous formulations of the Fourth Law of thermodynamics mentioned above.

## Data Availability

Data are contained within the article.

## Abbreviations

The following abbreviations are used in this manuscript:

LTS	Lyapunov Thermodynamic Stability
NSS	Nonequilibrium Steady States
PIT	Phenomenological Irreversible Thermodynamics
eq	equilibrium state
